# Case Report: Focusing on the unexpected: decision-making and embolisation of an unexpected intercostal artery guided by intraoperative transoesophageal echocardiography in persistent thoracic aortic false lumen perfusion and growth

**DOI:** 10.3389/fcvm.2026.1755914

**Published:** 2026-03-11

**Authors:** Audrey Mouche, Laurent Brisard, Blandine Maurel

**Affiliations:** 1 Department of Vascular and Cardiac Surgery, l’Institut du Thorax, CHU Nantes, Nantes University, Nantes, France; 2 Anesthesia and Critical Care Department, CHU Nantes, Nantes, France

**Keywords:** aortic dissection, embolization, endoleak, false lumen, transoesophageal echocardiography

## Abstract

Persistent false lumen perfusion of the remaining dissected aorta after open or endovascular aortic repair remains a major determinant of late aneurysmal growth and reintervention, often driven by complex and sometimes unexpected haemodynamic mechanisms. This case involves a 77-year-old man with progressive false lumen enlargement following staged hybrid repair of a chronic type A aortic dissection. Despite apparently successful gutter embolisation around a Candy Plug, intraoperative transoesophageal echocardiography (TEE) revealed persistent false lumen flow. Careful TEE assessment identified an unexpected proximal intercostal artery as the source of reperfusion. Selective catheterisation and coil embolisation of this artery were successfully performed under combined TEE and fusion imaging guidance, achieving immediate and durable exclusion. This case highlights the unique value of intraoperative TEE as a real-time haemodynamic imaging tool that can detect endoleaks missed by angiography and directly guide therapeutic decision-making in complex aortic dissections.

## Introduction

Persistent false lumen perfusion of the remaining dissected aorta is a major cause of late aneurysmal growth, reintervention, and mortality over time (1, 2). Despite advances in hybrid aortic repair and dedicated devices such as the Candy Plug, complete false lumen exclusion can be difficult to achieve due to complex haemodynamic mechanisms and collateral reperfusion pathways (3, 4). TEE is widely recognised as a key intraoperative imaging modality, not only for monitoring but also for providing real-time haemodynamic information. As shown by Fattori et al., TEE can detect residual flow and endoleaks not visualised by angiography, thereby influencing intraoperative decision-making (5).

We report a case of persistent false lumen enlargement, diagnosed on computed tomography angiography (CTA), following endovascular repair (TEVAR + Candy Plug) of a chronic post-type A aneurysmal residual dissection. During a complementary procedure of peri-Candy Plug gutter embolisation, intraoperative TEE unexpectedly identified persistent false lumen perfusion despite a satisfactory result on the completion DSA. This finding enabled identification of the feeding proximal intercostal artery, which was then embolised. To our knowledge, this is one of the very few reported cases in which TEE directly identified a persistent mechanism of false lumen perfusion, guiding decision-making and achieving complete false lumen thrombosis, thus highlighting its pivotal role in complex thoracic aortic endovascular interventions.

## Case report

A 77-year-old man with a history of surgical repair for Stanford type A aortic dissection in 2014 (ascending aortic replacement) was under regular follow-up. In early 2024, surveillance imaging showed progressive aneurysmal degeneration of the remaining dissected descending thoracic aorta, reaching a diameter of 60 mm. The patient was asymptomatic.

A staged hybrid approach was performed. First, in June 2024, a left carotid–subclavian artery transposition and a redo aortic arch replacement with a Thoraflex™ Hybrid prosthesis (Terumo Aortic, Inchinnan, Scotland, UK), with a distal stented length of 100 mm, were performed. In September 2024, endovascular coverage of a large entry tear in the mid-descending thoracic aorta was achieved with a Zenith Alpha™ Thoracic Endovascular Graft (Cook Medical, Bloomington, IN, USA), and embolisation of the false lumen was performed with deployment of a Candy Plug III (Cook Medical, Bloomington, IN, USA) sized to the false lumen diameter.

Despite these interventions, CTA in June 2025 showed aortic expansion up to 70 mm in diameter in the mid descending thoracic aorta and persistent partial perfusion of the false lumen through a gutter adjacent to the Candy Plug. Given the documented growth and persistent perfusion, a complementary embolisation procedure was planned in a hybrid operating room under fusion imaging guidance and TEE.

Gutter embolisation was attempted using Amplatzer™ Vascular Plug II devices (Abbott, Plymouth, MN, USA) and multiple detachable Ruby® Coils (Penumbra Inc., Alameda, CA, USA). Completion angiography suggested satisfactory exclusion of the peri–Candy Plug gutter, with no obvious residual opacification of the false lumen.

TEE, systematically performed to assess false lumen perfusion, demonstrated persistent, high-intensity colour Doppler flow within the false lumen, clearly distinct from low-velocity swirling flow or spontaneous echo contrast. Importantly, the spatial localisation and focal nature of the inflow identified on TEE were not consistent with residual peri–Candy Plug gutter perfusion, suggesting the presence of a distinct reperfusion mechanism.

TEE-guided assessment localised a focal inflow jet entering the false lumen at the level of a single proximal intercostal artery. Given the patient's prior extensive thoracic aortic coverage, the potential risk of spinal cord ischemia was carefully considered. The decision to proceed with embolisation was based on the combination of (i) documented aneurysmal growth on CTA, (ii) persistence of haemodynamically relevant false lumen perfusion despite angiographically satisfactory gutter embolisation, and (iii) identification of a discrete inflow source suitable for low-risk targeted embolisation. Standard spinal cord protection measures were applied, including strict maintenance of mean arterial pressure targets and close postoperative neurological monitoring.

Through prior embolisation access, a microcatheter was advanced to selectively catheterise this intercostal artery. Selective proximal embolisation was successfully performed using additional Ruby® Coils (Penumbra Inc., Alameda, CA, USA). Final intraoperative TEE confirmed immediate and complete cessation of false lumen flow, which was used as the procedural endpoint.

**Figure 1:**
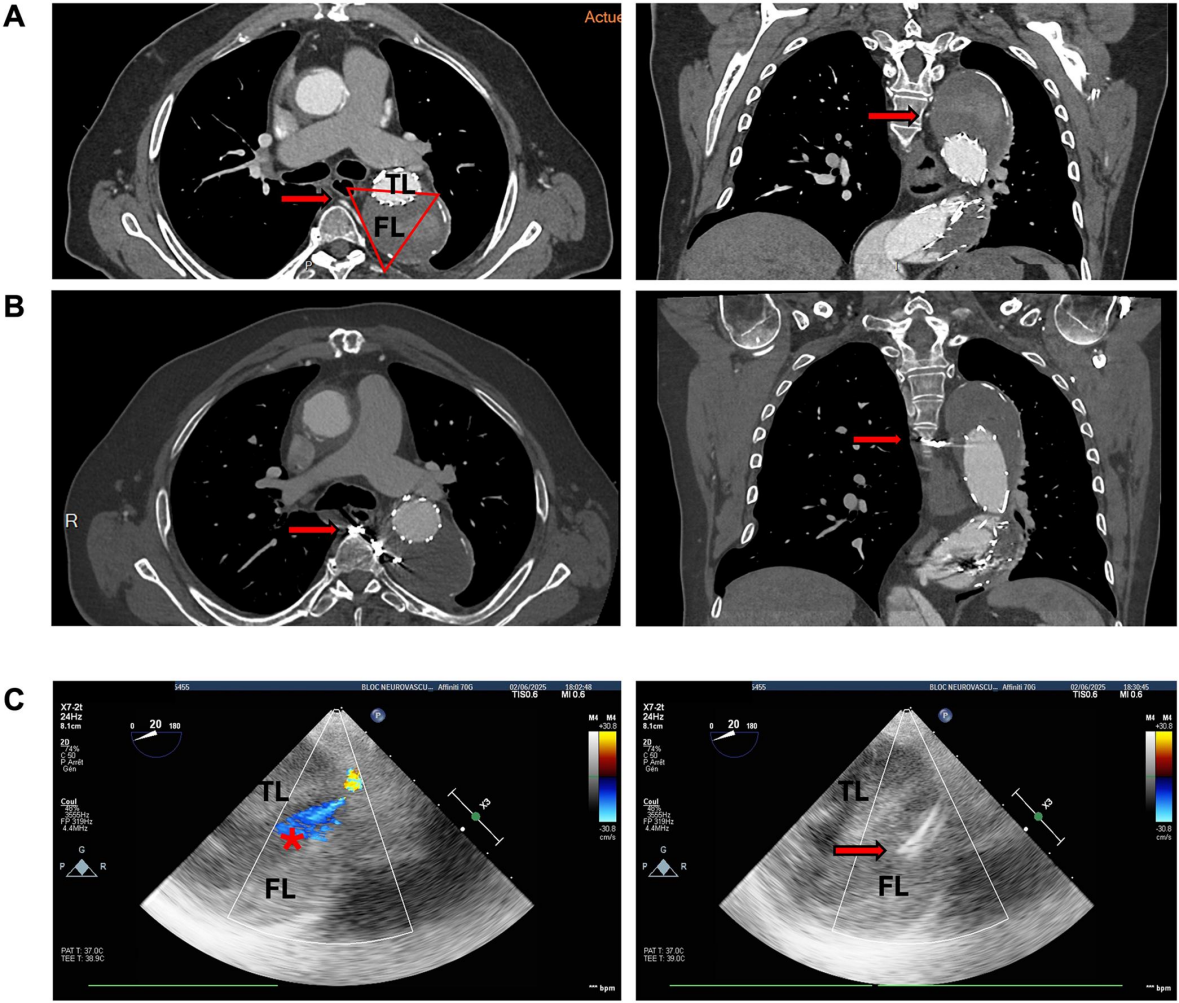
TEE-guided localisation and exclusion of an intercostal artery inflow at the T5 level: pre- and post-procedural CT and intraoperative imaging. **(A)** Pre-procedural contrast-enhanced computed tomography angiography (axial and coronal views) at the level of the fifth thoracic vertebra (T5), demonstrating persistent false lumen perfusion of the descending thoracic aorta related to a patent intercostal artery (red arrows). The red triangle indicates TEE probe orientation. The true lumen (TL) and false lumen (FL) are indicated. **(B)** Post-embolisation contrast-enhanced CT images at the same vertebral level (T5), showing microcoil material within the proximal intercostal artery (red arrows) with exclusion of false lumen perfusion. **(C)** Intraoperative transoesophageal echocardiography (TEE), short-axis views of the descending thoracic aorta at the corresponding T5 level. The TEE probe was positioned in the oesophagus at approximately 30 cm from the dental arch. The true lumen (TL), false lumen (FL). Top left: color Doppler demonstrates a focal inflow jet from the intercostal artery into the false lumen (red asterisk). Top right: coil artefact at the origin of the intercostal artery after selective embolisation (red arrow). Absence of residual colour Doppler signal within the false lumen, confirming complete flow cessation after embolisation.

Postoperative recovery was uneventful, with no neurological complications. At 5-month follow-up, CTA demonstrated durable thrombosis of the false lumen with no evidence of residual endoleak (Figure 1).

## Discussion

Persistent false lumen perfusion after endovascular repair of aortic dissection remains one of the major predictors of late aneurysmal growth and adverse outcomes. Despite advances in hybrid approaches and dedicated occlusion devices such as the Candy Plug, exclusion of the false lumen can be incomplete due to collateral reperfusion pathways (through intercostal or bronchial arteries) (4), target vessels dissection, or distal entry tears. Identifying these mechanisms intraoperatively is challenging, as completion angiography may underestimate residual flow.

In this case, progressive aneurysmal enlargement despite staged hybrid repair led to additional endovascular treatment targeting a peri–Candy Plug gutter identified on CTA. Although completion angiography suggested satisfactory exclusion, intraoperative transoesophageal echocardiography (TEE) demonstrated persistent haemodynamically relevant false lumen flow. TEE was not used independently to justify further intervention, but as a complementary haemodynamic tool integrated with documented aneurysmal growth, persistent perfusion, and procedural feasibility for low-risk targeted treatment.

The key contribution of TEE was the identification of a focal inflow mechanism not detected by angiography. The localisation and flow pattern were inconsistent with residual gutter perfusion and instead suggested collateral reperfusion via a proximal intercostal artery, a mechanism known to contribute to persistent false lumen perfusion in chronic dissections (4). Similar to previous reports, angiography alone underestimated residual haemodynamically relevant flow, whereas TEE provided real-time functional information that directly influenced decision-making (5).

Selective embolisation of intercostal arteries raises concerns regarding spinal cord ischemia, particularly in patients with extensive thoracic aortic coverage. In this case, embolisation was limited to a single, proximal intercostal artery identified as the dominant inflow source. Standard spinal cord protection measures were applied, and no neurological complications occurred, supporting the feasibility of highly targeted embolisation when clinically justified.

Beyond detection and localisation, TEE also defined the procedural endpoint by confirming immediate cessation of false lumen flow. This real-time confirmation may help avoid unnecessary additional embolization.

This report illustrates that intraoperative TEE can reveal clinically relevant false lumen reperfusion mechanisms missed by angiography and directly influence therapeutic decision-making in complex thoracic aortic repair. The main limitations are the single-case nature of the report and the mid-term follow-up.

## Data Availability

The raw data supporting the conclusions of this article will be made available by the authors, without undue reservation.

## References

[1] Erbel R Aboyans V Boileau C Bossone E Di Bartolomeo R Eggebrecht H 2014 ESC guidelines on the diagnosis and treatment of aortic diseases: document covering acute and chronic aortic diseases of the thoracic and abdominal aorta of the adult. The task force for the diagnosis and treatment of aortic diseases of the European Society of Cardiology (ESC). Eur Heart J. (2014) 35(41):2873–926. 10.1093/eurheartj/ehu281 25173340

[2] Evangelista A Isselbacher EM Bossone E Gleason TG Di Eusanio M Sechtem U Insights from the international registry of acute aortic dissection: a 20-year experience of collaborative clinical research. Circulation. (2018) 137(17):1846–60. 10.1161/CIRCULATIONAHA.117.031264 29685932

[3] Ogawa Y Nishimaki H Chiba K Murakami K Sakurai Y Fujiwara K Candy-plug technique using an excluder aortic extender for distal occlusion of a large false lumen aneurysm in chronic aortic dissection. J Endovasc Ther. (2016) 23(3):483–6. 10.1177/1526602816640523 27009975

[4] Rohlffs F Tsilimparis N Fiorucci B Heidemann F Debus ES Kölbel T. The candy-plug technique: technical aspects and early results of a new endovascular method for false lumen occlusion in chronic aortic dissection. J Endovasc Ther. (2017) 24(4):549–55. 10.1177/1526602817709252 28490232

[5] Fattori R Caldarera I Rapezzi C Rocchi G Napoli G Parlapiano M Primary endoleakage in endovascular treatment of the thoracic aorta: importance of intraoperative transesophageal echocardiography. J Thor Cardiovasc Surg. (2000) 120(3):490–5. 10.1067/mtc.2000.108904 10962409

